# Minimizing Radiation—Why Aren’t We Down to Zero?

**DOI:** 10.19102/icrm.2018.090807

**Published:** 2018-08-15

**Authors:** Alan Sugrue, Katherine Y. Le, Patrick Dearani, Samuel J. Asirvatham

**Affiliations:** ^1^Division of Heart Rhythm Services, Department of Cardiovascular Diseases, Mayo Clinic, Rochester, MN, USA; ^2^Division of Pediatric Cardiology, Department of Pediatric and Adolescent Medicine, Mayo Clinic, Rochester, MN, USA

**Keywords:** Augmented reality, radiation, virtual reality, zero fluoroscopy

## Introduction

Radiation exposure is a real concern not only in the medical field but also for laypeople and society in general. It seems that there are monthly, if not weekly, news articles detailing concerns regarding nonionizing (eg, mobile phones, wireless transmission) or ionizing radiation (eg, tanning beds, medical X-rays). In the electrophysiology (EP) sphere, radiation is of particular concern given the field’s historical reliance on fluoroscopy for guiding catheter manipulation and for defining anatomical landmarks for cardiac ablation or device implantation. Radiation exposure is predicted to increase with exponential growth in indications for procedures (both ablation and cardiac devices) and a rise in the complexity of cases, thus increasing the time of exposure. With the recognized and growing understanding of adverse effects from radiation exposure on the operator, medical staff, and patient, minimizing radiation has become a vital element of procedural care. Although there are no reservations that excessive radiation can cause significant biological harm and there is no safe level of radiation exposure, the problem in current practice is determining how can this risk be best mitigated and, equally significant, what tradeoffs occur when one attempts to reduce this risk.

## “Zero” or “near-zero” fluoroscopy

The concept of performing an EP procedure without fluoroscopy is intuitively attractive for the patient, the proceduralist, and the laboratory staff. The idea of zero/near-zero fluoroscopy was first reported in 2002^1^ and cultivated from the desire and need to minimize radiation exposure in EP procedures performed in children and pregnant women. Success in these groups of patients has driven the expanded application of this approach across a range of populations, ages, and procedures.^2,3^ Without fluoroscopy, two significant alternatives can help in the localization and guidance of catheters: intracardiac echocardiogram (ICE) and three-dimensional (3D) electroanatomical mapping (EAM) systems. However, although offsetting or minimizing fluoroscopy is advantageous, the proceduralist and the patient must consider the significant tradeoff of employing this approach. First, is there an increased risk of harm/complications for the patient? More specifically, is there an increased risk for perforation, incorrect ablation, and/or damage to collateral structures?^4^ With the historical dependence and experience gained from years of fluoroscopy, one may instinctively feel this would be the case because, with fluoroscopy, one has learned to predict catheter movement. However, a meta-analysis of 10 clinical studies involving 2,261 patients reported that safety with a zero/near-zero fluoroscopic approach was similar to the standard two-dimensional fluoroscopic approach,^5^ with more recent studies also endorsing a similar safety profile.^3,6^ Although the overall number of patients reported is relatively small, it is generally reassuring that there seems to be no increased risk from zero or near-zero fluoroscopic approaches.

While the safety data are reassuring, the second important question to consider is regarding efficacy—more specifically, does the use of alternative systems (eg, 3D EAM mapping, ICE) affect ablation, intraprocedural device success, and/or long-term outcomes? First, by focusing intently on a near/near-zero approach, we may be limiting opportunities for fine/more-detailed mapping (although this concern has become slightly offset by the growth and advancements of EAM catheters and technology). Second, proceduralists may find the catheter in an unusual or not readily recognizable location, and with no fluoroscopy to help reassure them, they may thus be less likely to deliver therapy in these areas. Nevertheless, data on efficacy are reassuring and overall promising,^7,8^ suggesting that there is minimal to no impact on procedural or long-term outcomes. Further data will be required to support these preliminary findings definitely, and this will be important, as this approach is employed in more complicated procedures.

The literature seems to suggest that, overall, there does not seem to be any significant tradeoff with not using fluoroscopy, particularly regarding the safety and efficacy profile. To this end, then, why have we not seen a complete transition to the use of zero/near-zero fluoroscopy? One significant consideration for the adoption of any new technology or approach is cost. The use of EAM and ICE adds significant cost to the procedure. Although no formal cost analysis has been performed, preliminary estimates suggest that the increase in life expectancy and period of life without cancer attributed to a minimally fluoroscopic approach is economically affordable.^9^ Of note, also, many EP laboratories have both EAM and ICE embedded in their day-to-day practice, which generally represent the backbone of atrial fibrillation and atrial flutter cases for both mapping and transseptal access. Therefore, with these systems already present, there seems to be limited further financial implications for centers and patients. In addition to cost, however, there are some definite limitations of EAM and ICE that may be contributing to the continued use of fluoroscopy. First, aside from the technical limitations of EAM on a broader scale and the time required to create the maps, EAM relies entirely on reference stability both from the patient and the intracardiac reference catheter. Further, the created anatomical maps may not be completely accurate, but it does a significantly better job than fluoroscopy, where anatomical landmarks are based upon shadows, catheter position, and movement. Regarding ICE, images are sector-based and it can be difficult to reconstitute 3D views. Lastly, there is a significant learning curve^10^ for these new systems. Although this learning curve is diminishing as more people gain experience with these systems, initially, there may be continued reliance on fluoroscopy to help with familiarity.

Though these limitations are essential considerations in the slow adaption of zero/near-zero fluoroscopy, we believe that the additional primary driver in the ongoing use of fluoroscopy is habit formation and the physiological aspects of medical practice in general, especially EP. EP is in a unique situation in that it is a relatively new field, with its growth and expansion occurring within the active professional life of most EP doctors. Subsequently, many learned using fluoroscopy—ie, they were taught with it and then they taught others with it. It has become so embedded and almost second nature that it can be difficult to change this dependence. A wide range of psychosocial factors influence the way health-care professionals practice their craft as well as their willingness to adopt change in clinical practice.^11^ Social cognitive theories have been utilized to understand the thought processes behind clinicians’ intentions and behaviors.^12–14^ Of the social cognitive theories proposed, the theory of reasoned action^15^ best describes this lack of implementation. This theory suggests that a person’s behavior is determined by their intention to perform the action. This intention is a function of two determinants: (1) attitude toward the behavior and (2) subjective norms. Of these two determinants, subjective norms (social influence) likely plays the largest role, as the historical reliance on fluoroscopy creates a norm that its use is standard. As EP continues to grow and both attitude (conscious and subconscious) and social norms toward fluoroscopy change and evolve, we will likely see the further widespread adoption of zero/near-zero fluoroscopy.

It is within this context that Rogers and Brodt^16^ provide a well-written and important narrative review that discusses the potential hazards of ionizing radiation exposure, general approaches for decreasing fluoroscopy, and specific task-based alternatives to fluoroscopy. This is a must-read for a cardiologist and especially the proceduralist. The article is composed of two sections: a general approach and a specific task section. In the general approach section, methods to reduce fluoroscopy are succinctly described. As the authors state, these general measures represent low-hanging fruit, as they do not require equipment upgrades, specialized training, or significant changes to the procedural workflow; these are essentially techniques that can be implanted in the laboratory very quickly and should be standard practice. However, the widespread adoption of these techniques remains low. The review continues on to highlight specific procedures/interventions that operators can employ to minimize radiation dosing. One example is transseptal puncture (TP). TP, which has historically relied on fluoroscopy, can be safely performed instead with ICE guidance, which enables clear delineation of the transseptal needle location relative to other cardiac structures and can also enable characterization of the interatrial septum.

The authors additionally do a thorough job of highlighting different technologies available to help reduce fluoroscopy; however, there is one other category that we would like to also mention, which is virtual reality (VR) and augmented reality (AR). We are currently at the beginning of the integration of both VR and AR into our everyday lives, and the same applies to these technologies’ adoption into medicine. For example, in February of this year, the National Institutes of Health (Bethesda, MD, USA) awarded a $2.2-million research grant to SentiAR Inc. (St. Louis, MO, USA) to design AR software to improve visualization in cardiac surgeries and other interventional procedures. The hope is that this technology will ultimately enable physicians to view, measure, and manipulate real-time holographic images of the patient’s heart during medical procedures, while still being able to see the operating room environment. This will remarkably improve physicians’ complete, real-time, and visual control of both the virtual and real worlds. Even more recently, an AR approach to guide epicardial needle puncture by projecting the patient-specific 3D anatomy onto the procedural environment was proposed and is due to undergo testing.^17^ As we see the growth and evolution of these technologies, such developments will not only raise new challenges but will also bring about a new generation of change to the EP world. VR and AR may represent the advance that finally pushes fluoroscopy out the door.
